# Prevalence of *RECQL* germline variants in Pakistani early-onset and familial breast cancer patients

**DOI:** 10.1186/s13053-020-00159-6

**Published:** 2020-12-20

**Authors:** Muhammad Usman Rashid, Noor Muhammad, Faiz Ali Khan, Umara Shehzad, Humaira Naeemi, Naila Malkani, Ute Hamann

**Affiliations:** 1 Department of Basic Sciences Research, Shaukat Khanum Memorial Cancer Hospital and Research Centre (SKMCH&RC), 7A, Block R3, Johar Town, Lahore, Punjab 54000 Pakistan; 2 Molecular Genetics of Breast Cancer, German Cancer Research Center (DKFZ), Im Neuenheimer Feld 580, 69120 Heidelberg, Germany; 3 Department of Zoology, Government College University, Lahore, Pakistan

**Keywords:** *RECQL*, Germline variants, Breast cancer, Pakistan

## Abstract

**Background:**

The RecQ Like Helicase (*RECQL*) gene has previously been shown to predispose to breast cancer mainly in European populations, in particular to estrogen receptor (ER) and/or progesterone receptor (PR) positive tumor. Here, we investigated the contribution of pathogenic *RECQL* germline variants to hereditary breast cancer in early-onset and familial breast cancer patients from Pakistan.

**Methods:**

Comprehensive *RECQL* variant analysis was performed in 302 *BRCA1* and *BRCA2* negative patients with ER and/or PR positive breast tumors using denaturing high-performance liquid chromatography followed by DNA sequencing. Novel variants were classified using Sherloc guidelines.

**Results:**

One novel pathogenic protein-truncating variant (p.W75*) was identified in a 37-year-old familial breast cancer patient. The pathogenic variant frequencies were 0.3% (1/302) in early-onset and familial breast cancer patients and 0.8% (1/133) in familial patients. Further, three novel variants of unknown significance, p.I141F, p.S182S, and p.C475C, were identified in familial breast cancer patients at the age of 47, 68, and 47 respectively. All variants were absent in 250 controls.

**Conclusions:**

Our data suggest that the *RECQL* gene plays a negligible role in breast cancer predisposition in Pakistan.

## Background

Globally, the incidence of breast cancer has increased to approximately 2 million cases in 2017, while the mortality rate between 2007 and 2017 has declined [1]. In Pakistan, breast cancer is the most frequent invasive malignancy among women, accounting for 36.8% of all female malignancies [2]. Pakistan has one of the highest rates of breast cancer in Asia, with age-standardized (world) annual incidence and mortality rates of 43.9 and 23.2 per 100,000, respectively [2]. Breast cancer incidence and mortality trends are still increasing [3, 4], making breast cancer a major public health burden in this developing country.

Approximately 50% of familial breast cancer is due to pathogenic germline variants in high, and moderate penetrance genes and common low-penetrance genetic variants [5]. Most of these genes are involved in the DNA repair pathway and maintenance of genomic stability, underlining the significance of other genes involved in this pathway. In 2015, the RecQ Like Helicase (*RECQL*) gene was identified in West European and East Asian populations as a candidate breast cancer susceptibility gene [6, 7]. It encodes a DNA helicase, which is involved in the repair of DNA double-strand breaks and plays a crucial role in the maintenance of genomic stability. Several studies conducted among unselected breast cancer patients from Belarus and Germany [8], USA [9], and early-onset and familial breast cancer patients from Poland [6], Canada [6], and Australia [10] reported pathogenic *RECQL* variant frequencies ranging from 0 to 2.6%. Breast tumors associated with pathogenic *RECQL* variants were predominantly positive for the estrogen and progesterone receptors (ER and PR) [6–8, 11].

Apart from two studies conducted in an East Asian population from China [7, 11], data on the contribution of pathogenic *RECQL* variants to early-onset and/or familial breast cancer patients from other Asian regions are lacking. In Pakistan, breast cancer is the most common malignancy and main cause of cancer-related deaths in women. The burden of breast cancer in terms of estimated age-standardised incidence and mortality rates is 43.9 and 23.2 per 100,000, respectively [12]. Pathogenic variants in high- and moderate-penetrance breast cancer susceptibility genes (*BRCA1*, *BRCA2*, *TP53, CHEK2*, *RAD51C,* and *PALB2*) account for about 27% of early-onset and familial breast cancers in Pakistan [13–17], leaving a substantial proportion of cases unexplained. In the present study, we determined the contribution of pathogenic *RECQL* variants to hereditary breast cancer in 302 early-onset and familial *BRCA1* and *BRCA2* negative patients with ER positive and/or PR positive breast cancer in a South Asian population from Pakistan.

## Methods

### Study subjects

Patients diagnosed with invasive breast cancer were selected from the institutional registry of genetically enriched breast and ovarian cancer families enrolled at the Shaukat Khanum Memorial Cancer Hospital and Research Centre (SKMCH&RC) in Lahore, Pakistan, from June 2001 to August 2015, fulfilling the inclusion criteria as described previously [17, 18]. The present study included 302 early-onset and familial breast cancer patients with ER positive and/or PR positive tumors. All study participants were tested negative for pathogenic variants in *BRCA1, BRCA2* [17, 18] and about 60% for pathogenic variants in *PALB2* (*n* = 187), *TP53* (*n* = 180), *CHEK2* (*n* = 168), and *RAD51C* (n = 168) [13–16] (Muhammad U. Rashid, unpublished *TP53* data). We categorized study participants into four risk groups based on age at cancer diagnosis or family history of breast and/or ovarian cancer (Table 1) [17].

**Table 1:** Frequency of *RECQL* pathogenic variants according to family structure

Risk group	Phenotype of families	No. of families	Families with *RECQL* pathogenic variant n (%)
	All families	302	1 (0.3)
A1 + A2 + A3	Female breast cancer families	255	1 (0.4)
A1	Early-onset breast cancer (1 case ≤30 years)	122	0 (0.0)
A2 + A3	Familial breast cancer (≥2 cases, ≥1 diagnosed ≤50 years)	133	1 (0.8)
A4	Male breast cancer (1 case diagnosed at any age)	29	0 (0.0)
B	Breast and ovarian cancer families (≥1 breast cancer and ≥ 1 ovarian cancer)	18	0 (0.0)

The control population comprised 250 healthy women with no family history of breast/ovarian cancer. They were selected from the institutional registry of 1012 female controls enrolled in a Pakistani breast cancer case-control study as previously described [19]. The Institutional Review Board (IRB) of the SKMCH&RC approved the current study (IRB approval number ONC-BRCA-001/2). All study participants signed informed written consent.

### Variant screening

The complete coding sequence and exon-intron junctions of the *RECQL* gene (Genbank accession number NM_002907.3) were screened in the 302 index patients and 250 controls by denaturing high-performance liquid chromatography (DHPLC) analysis. The PCR primers details are described elsewhere [7]. When available, a positive control with a known variant was included in each set of DHPLC analysis. Bidirectional DNA sequencing was performed to confirm a variant, as described elsewhere [20].

### Variant classification

The novel *RECQL* variants were analyzed using the numerical score-based variant classification system Sherloc, a comprehensive refinement of the American College of Medical Genetics and Genomics-Association for Molecular Pathology (ACMG-AMP). Five evidence categories (two clinical and three functional) were used to evaluate variants. Clinical criteria include variant frequency information from large human population data, the Genome Aggregation Database (gnomAD; https://gnomad.broadinstitute.org/gene/-ENSG00000004700?dataset=gnomad_r2_1) and variant observation in unaffected and affected individuals and families. For variants classification, allele frequencies of South Asian population from gnomAD were used as this population has ethnic and geographic relevance to Pakistani population. Functional criteria include variant type, experimental studies, and computational data. The following in silico tools for prediction of protein function or splicing were used: PolyPhen-2 (http://genetics.bwh.harvard.edu/pph2/), SIFT (https://sift.bii.a-star.edu.sg/), SNAP2 (http://www.rostlab.org/services/snap/submit), MutationTaster (http://www.mutationtaster.org/), SNPs&GO (http://snps.biofold.org/snps-and-go/snps-and-go.html), and nsSNP Analyzer (http://snpanalyzer.uthsc.edu/) for the missense variants, [14, 16] and splice-site prediction algorithms MaxEntScan (http://genes.mit.edu/burgelab/maxent/Xmaxentscan_scoreseq.html), NNSPLICE (http://www.fruitfly.org/seq_tools/splice.html), HumanSplice Finder (http://www.umd.be/HSF3/), GeneSplicer (http://ccb.jhu.edu/software/genesplicer/), and SpliceSiteFinder-like (http://www.umd.be/searchSpliceSite.html) for splice-site and intronic variants [14]. In case of any disagreement between clinical and functional evidence, the clinical evidence was considered more convincing.

Variants were classified as pathogenic, likely pathogenic, benign, likely benign, and as variants of uncertain significance (VUS), according to the Sherloc guidelines [21]. Sherloc is a semiquantitative system in which each criterion is awarded a preset number of points on orthogonal benign (1B-5B) or pathogenic (1P-5P) scales using clinical and functional criteria. Point thresholds for pathogenic and benign classifications are 5P and 5B, for likely pathogenic and likely benign classifications 4P and 3B, and for VUS <4P and < 3B. Pathogenicity and benign point scores were calculated separately.

### RNA analysis of the c.868-2A > G splice-site variant

Total RNA was extracted from blood samples of the proband and an unaffected sister harboring the *RECQL* c.868-2A > G, another variant negative unaffected sister, and a variant negative control using TRIzol reagent (Invitrogen, Carlsbad, CA, USA). Total RNA was transcribed to cDNA using the RevertAid First Strand cDNA Synthesis Kit (Thermo Fisher Scientific, Vilnius, Lithuania) with random hexamer primers according to the manufacturer’s protocol. Reverse transcriptase (RT)-PCR was performed using the forward primer (5′ – CAG TTC CCT AAC GCA TCA CT – 3′) and reverse primer (5′ – TTT CAT TGG CTG ACC ATT TT – 3′) located on exon 7 and exon 9 of the *RECQL* transcript variant 1 (ENST00000444129.7), respectively. PCR reactions were carried out in a 25 μl volume containing 1 μl of respective cDNA, 1x PCR Gold Buffer (Applied Biosystems, California, USA), 2.5 mM MgCl_2_, 0.2 μM of each primer, 250 μM of each dNTP (Invitrogen, Carlsbad CA, USA), and 1 unit AmpliTaq Gold DNA polymerase (Applied Biosystems, California, USA). After an initial denaturation for 15 min at 95 °C, cDNA was amplified by 35 cycles of 1 min at 94 °C, 1 min at 57.5 °C, 1 min at 72 °C, and a final extension step of 5 min at 72 °C. Five μl of RT-PCR products were loaded on a 2% agarose gel containing ethidium bromide (Sigma-Aldrich, Steinheim, Germany) and electrophoresis was performed at 140 V for 80 min and confirmed by Sanger sequencing as described previously [20].

## Results

### Characteristics of the study participants

A total of 302 *BRCA1* and *BRCA2* negative index breast cancer patients were screened for *RECQL* germline variants. Of these patients, 122 (40.4%) were early-onset breast cancer patients (≤30 years of age), 133 (44.0%) belonged to families with two or more breast cancer cases with at least one case diagnosed at 50 years or younger, 18 (6.0%) to families with both breast and ovarian cancer, and 29 (9.6%) male breast cancer cases diagnosed at any age (Table 1). Of the index patients, 223 presented with ER positive and PR positive breast tumors, 55 with ER positive tumors, and 24 with PR positive tumors. The mean age of disease presentation was 36.6 years (range 20–78) for female breast cancer (*n* = 273), and 51.5 years (range 27–73) for male breast cancer (*n* = 29).

### Spectrum of identified *RECQL* variants

In total, 31 distinct *RECQL* variants were detected. Of these, 20 were novel: one nonsense variant, one splice-site variant, three missense variants, three silent variants, and twelve noncoding variants (Table 2). The remaining eleven variants were previously reported: three missense variants and eight noncoding variants.

**Table 2:** *RECQL* germline variants identified in the study cases and controls from Pakistan

Location	Coding (c.) DNA Sequence^a^	Effect	Prevalence n (%)		MAF (%)			Classification	Reference
			Cases (*n* = 302)	Controls (*n* = 250)	Cases	Controls	gnomAD, SAS		
*Pathogenic variant*									
Exon 4	c.225G > A (p.W75*)	Nonsense	1 (0.3)	0 (0.0)	0.166	0	NA	P^b^	Novel
*Variants of uncertain significance*									
Exon 5	c.421A > T (p.I141F)	Missense	1 (0.3)	0 (0.0)	0.166	0	0.0188	VUS^b^	Novel
Exon 6	c.546C > T (p.S182S)	Silent	1 (0.3)	0 (0.0)	0.166	0	0.0165	VUS^b^	Novel
Exon 12	c.1425C > T (p.C475C)	Silent	1 (0.3)	0 (0.0)	0.166	0	0.0033	VUS^b^	Novel
*Benign variants*									
*Coding*									
Exon 3	c.132G > A (p.K44K)	Silent	34 (11.3)	28 (11.2)	5.629	5.6	NA	B	Novel
Exon 3	c.151G > A (p.E51K)	Missense	49 (16.2)	34 (13.6)	8.113	6.8	0.0098	B	Novel
Exon 7	c.833C > G (p.T278R)	Missense	5 (1.7)	6 (2.4)	0.828	1.2	1.6417	B	ClinVar, [10]
Exon 8	c.898 T > A (p.F300I)	Missense	1 (0.3)	0 (0.0)	0.166	0	0.0033	B	[6, 10]
Exon 13	c.1651A > G (p.I551V)	Missense	1 (0.3)	1 (0.4)	0.166	0.2	0.0785	LB^b^	Novel
Exon 13	c.1661A > G (p.Y554C)	Missense	5 (1.7)	1 (0.4)	0.828	0.2	0.2366	LB	[10]
*Non-coding variants*									
5’UTR	c.-110G > A	5’UTR	2 (0.7)	3 (1.2)	0.331	0.6	NA	B	Novel
5’UTR	c.-137C > T	5’UTR	3 (1.0)	3 (1.2)	0.497	0.6	NA	B	ClinVar, [22]
5’UTR	c.-187 T > G	5’UTR	1 (0.3)	0 (0.0)	0.166	0	NA	B	Novel
Intron 3	c.215-169C > A	Intronic	22 (7.3)	20 (8.0)	3.642	4.0	0	LB	ClinVar
Intron 3	c.215-86G > A	Intronic	1 (0.3)	1 (0.4)	0.166	0.2	0	B	[22]
Intron 3	c.215-48C > A	Intronic	1 (0.3)	1 (0.4)	0.166	0.2	0.0663	B	Novel
Intron 3	c.215-37 T > C	Intronic	1 (0.3)	0 (0.0)	0.166	0	0.0174	B	Novel
Intron 6	c.700 + 110C > G	Intronic	1 (0.3)	0 (0.0)	0.166	0	NA	B	Novel
Intron 7	c.868-11G > A	Intronic	1 (0.3)	1 (0.4)	0.166	0.2	0.0611	B	Novel
Intron 7	c.868-2A > G	Intronic	3 (1.0)	2 (0.8)	0.497	0.4	0.5669	B^b^	Novel
Intron 8	c.949 + 62A > G	Intronic	1 (0.3)	0 (0.0)	0.166	0	NA	B	Novel
Intron 8	c.949 + 76A > G	Intronic	6 (2.0)	6 (2.4)	0.993	1.2	0	LB	ClinVar
Intron 11	c.1355 + 30 T > C	Intronic	13 (4.3)	17 (6.8)	2.152	3.4	51.4	B	[22]
Intron 11	c.1355 + 103G > C	Intronic	5 (1.7)	11 (4.4)	0.828	2.2	0	B	ClinVar, [22]
Intron 12	c.1448-18A > G	Intronic	1 (0.3)	0 (0.0)	0.166	0	0	B	Novel
Intron 13	c.1667 + 53 T > A	Intronic	2 (0.7)	1 (0.4)	0.331	0.2	0	B	ClinVar
Intron 13	c.1667 + 53delT	Intronic	1 (0.3)	0 (0.0)	0.166	0	0	B	Novel
Intron 13	c.1668-160C > T	Intronic	1 (0.3)	0 (0.0)	0.166	0	0	B	Novel
Intron 13	c.1668-81G > A	Intronic	3 (1.0)	4 (1.6)	0.497	0.8	0	B	Novel
Intron 14	c.1797 + 14_17delAATT	Intronic	20 (6.6)	22 (8.8)	3.311	4.4	4.0024	B	Novel
3’UTR	c.*6A > C	3’UTR	61 (20.2)	84 (33.6)	10.099	16.8	NA	B	[22]

*B* Benign, *gnomAD* Genome aggregation database, *LB* Likely benign, *LP* Likely pathogenic, *MAF* Minor allele frequency, *NA* Not available, *P* Pathogenic, *SAS* South Asians, *VUS* Variant of uncertain significance

^a^Nomenclature follows Human Genome Variation Society (HGVS) (http://www.hgvs.org). Numbering start at the first A of the first coding ATG (located in exon 2) of NCBI GenBank Accession NM_002907.3

^b^Classification of nucleotide alterations was performed using Sherloc guidelines [21]

### Classification and characteristics of identified *RECQL* variants

The novel variants were analyzed for their potential functional effect using Sherloc guidelines [21], including the minor allele frequency (MAF) > 1% for benign variants reported in Genome Aggregation Database (gnomAD) or in our study (Table 2) and in silico prediction tools (Table 3). One variant was classified as pathogenic, three as VUS, and 16 as benign/likely benign.

**Table 3:** In silico analyses of novel *RECQL* variants identified in the study cases from Pakistan

Coding variants	In silico prediction						Consensus^a^
	PolyPhen-2	SIFT	SNAP2	MutationTaster	SNPs&GO	nsSNP Analyzer	
c.151G > A (p.E51K)	Benign	Tolerated	Neutral	Disease causing	Neutral	Neutral	Benign
c.421A > T (p.I141F)	Probably damaging	Deleterious	Effect	Disease causing	Disease	Disease	Deleterious (6/6)
c.1651A > G (p.I551V)	Benign	Tolerated	Neutral	Polymorphism	Neutral	Neutral	Benign
Noncoding variants	Splice-site predictions						Consensus^a, b^
	SpliceSiteFinder-like	MaxEntScan	NNSPLICE	GeneSplicer	HumanSplice Finder		
c.-110G > A	NE	NE	NE	NE	NE		Benign
c.-187 T > G	D (0 → 73.0)	D (0 → 2.9)	NE	NE	NE		Benign
c.215-48C > A	NE	NE	NE	NE	NE		Benign
c.215-37 T > C	NE	NE	NE	NE	NE		Benign
c.700 + 110C > G	NE	NE	NE	NE	A (0 → 80.1)		Benign
c.868-11G > A	A (0 → 85.8)	A (2.5 → 7.1)	NE	NE	NE		Benign
c.868-2A > G	A (0 → 79.9)^c^	A (0 → 5.4)^c^	A (0 → 0.4)^c^	NE	NE		Deleterious (3/5)
c.949 + 62A > G	NE	NE	NE	NE	NE		Benign
c.1448-18A > G	NE	NE	NE	NE	NE		Benign
c.1667 + 53delT	NE	NE	NE	NE	NE		Benign
c.1668-160C > T	NE	D (2.9 → 1.2)	NE	NE	NE		Benign
c.1668-81G > A	NE	NE	NE	NE	NE		Benign
c.1797 + 14_17delAATT	NE	A (4.8 → 2.3)	NE	NE	NE		Benign

*A* Acceptor, *D* Donor, *NE* No effect

^a^The variant is considered as deleterious by six of the six protein function prediction or three of the five splice-site prediction algorithms for coding or noncoding variants, respectively

^b^ > 20% change in score (i.e., a wild-type splice-site score decreases and/or a cryptic splice-site score increases) is considered significant

^c^Canonical splice acceptor site is abolished (MaxEntScan score + 2.46 → -5.49) and creates a cryptic splice acceptor site at c.877

### Pathogenic *RECQL* variant

The novel pathogenic *RECQL* variant is a nonsense variant at nucleotide position 225 in exon 4 (c.225G > A (p.W75*)), which is predicted to result in premature protein termination. It was identified in a 37-year-old familial breast cancer patient (III:3, Fig. 1a) of Punjabi ethnicity and was absent in 250 controls. The patient carrying this variant presented with a grade 3, ER positive and PR positive invasive ductal carcinoma (IDC) with lymph node involvement. The pathogenic variant frequencies were 0.3% (1/302) in early-onset and familial breast cancer patients and 0.8% (1/133) in familial patients. The variant had a Sherloc score of 8P and was classified as pathogenic (Table 4).

**Fig. 1:**
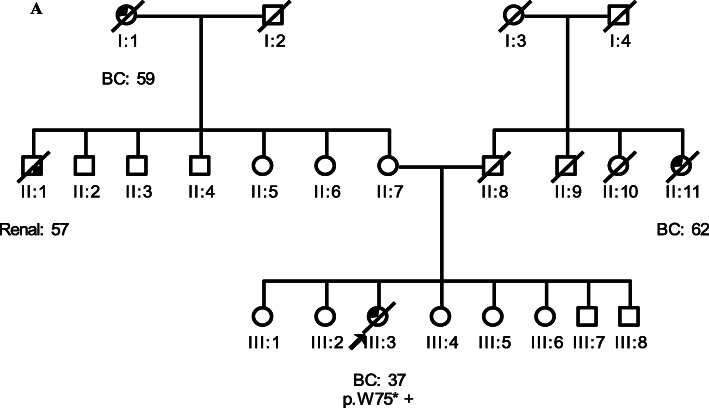
Pedigrees of breast cancer patients with *RECQL* variants. **a** Family 282 carrying the pathogenic variant p.W75*. **b-d** Families 565, 649, and 625 carrying the VUS p.I141F, p.S182S, and p.C475C, respectively. **e-g** Families 471, 577 and 595 carrying the benign variant c.868-2A > G. *Circles* are females, *squares* are males, and a *diagonal slash* indicates a deceased individual. *Symbols* with filled *left upper* quadrant: unilateral breast cancer. *Symbols* with *filled right lower* quadrant: cancer other than breast, the name of that cancer is indicated. *Double line* between spouses: consanguineous marriage. Identification numbers of individuals are below the *symbols*. The index patient is indicated by an *arrow*. *BC:* breast cancer. The numbers following these abbreviations indicate age at cancer diagnosis. +: carrier, −: non-carrier

**Table 4:** Sherloc variant classification criteria of novel *RECQL* variants

Variant	Evidence #	P/B	Points score^a^	Evidence type	Category
c.225G > A (p.W75*)	EV0135	P	1	Clinical	Population - Frequency
	EV0211	P	0	Clinical	Observation in Affecteds
	EV0206	P	2	Clinical	Observation in Affecteds
	EV0016	P	5	Functional	Variant Effect
	**Sum**		**8P**		
	**Sherloc score**		**Pathogenic**		
c.421A > T (p.I141F)	EV0101	P	0.5	Clinical	Population - Frequency
	EV0211	P	0	Clinical	Observation in Affecteds
	EV0172	P	1	Functional	Variant Effect
	EV0121	P	1	Functional	Computational & Predictive
	**Sum**		**2.5P**		
	**Sherloc score**		**VUS**		
c.546C > T (p.S182S)	EV0101	P	0.5	Clinical	Population - Frequency
	EV0211	P	0	Clinical	Observation in Affecteds
	EV0193	P	1	Clinical	Observation in Affecteds
	EV0103	B	2	Functional	Variant Effect
	EV0191	B	1	Functional	Computational & Predictive
	**Sum**		**1.5P, 3B**		
	**Sherloc score**		**VUS**		
c.1425C > T (p.C475C)	EV0101	P	0.5	Clinical	Population - Frequency
	EV0211	P	0	Clinical	Observation in Affecteds
	EV0193	P	1	Clinical	Observation in Affecteds
	EV0103	B	2	Functional	Variant Effect
	EV0191	B	1	Functional	Computational & Predictive
	**Sum**		**1.5P, 3B**		
	**Sherloc score**		**VUS**		
c.868-2A > G	EV0096	B	5	Clinical	Population - Frequency
	EV0053	B	2	Clinical	Observation in Unaffected
	EV0037	B	1	Functional	Functional Experiment
	EV0187	P	1	Functional	Computational & Predictive
	**Sum**		**1P, 8B**		
	**Sherloc score**		**Benign**		

*B* Benign, *EV* Evidence, *P* Pathogenic, *VUS* Variant of unknown significance

Pathogenicity and benign point scores are calculated separately

^a^The Sherloc point score thresholds for pathogenic and benign classifications are 5P and 5B, and for VUS <4P and < 3B

### *RECQL* variants of uncertain significance (VUS)

One novel missense variant (p.I141F) was identified in a 47-year-old familial breast cancer patient (II:4, Fig. 1b) of Punjabi origin. Two silent variants (p.S182S and p.C475C) were detected in familial breast cancer patients at age 68 (I:1, Fig. 1c) and 47 (III:10, Fig. 1d) respectively of Saraiki background. These variants were not detected in 250 controls. The population allele frequencies of p.I141F, p.S182S, and p.C475C were low (MAF = 0.0188%, MAF = 0.0165% and MAF = 0.0033%, respectively) and within the pathogenic range of < 8 total alleles among South Asians (*n* ≥ 12,086) in the gnomAD. The missense variant had a Sherloc score of 2.5P and both silent variants of P1.5 and B3. All variants were classified as VUS (Table 4).

### Benign or likely benign variants

One novel variant in a canonical splice acceptor site of intron 7, c.868-2A > G, was detected in a 36-year-old familial (II:4, Fig. 1e), a 61-year-old male (II:8, Fig. 1f), and a 25-year-old female early-onset breast cancer patient (II:9, Fig. 1g) of Punjabi, Urdu speaking and Pathan ethnicity, respectively (1%, 3/302). It was also found in one of the two tested unaffected sisters (II:7, Fig. 1g) of the early-onset patient. Moreover, c.868-2A > G was detected in two controls (0.8%, 2/250). The similar frequencies in cases and controls indicate that this variant is not likely to be pathogenic. Using the Sherloc guidelines, a high frequency of the G allele (MAF = 0.5669%) was reported among South Asians (*n* = 13,582) in the gnomAD. It was predicted to have a functional impact by three of five splice-site prediction tools (Table 3).

**Fig. 2:**
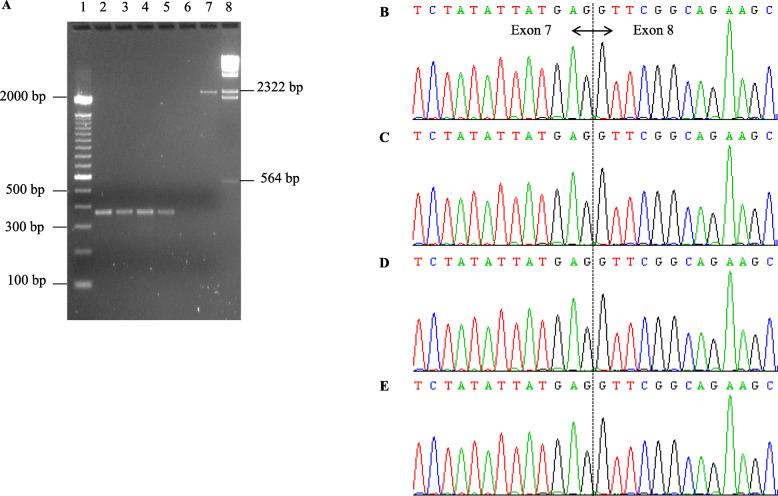
RT-PCR analysis of the *RECQL* c.868-2A > G splice-site variant. **a** Photograph of an ethidium bromide-stained gel of the *RECQL* transcripts. Lane 1, DNA 100 bp marker; lanes 2 and 3, c.868-2A > G carriers; lanes 4 and 5, non-carriers (family member, control); lane 6, no template control; lane 7, gDNA wild type control; lane 8, Lambda DNA/HindIII marker. Product sizes: gDNA = 2260 bp; cDNA = 364 bp. Sequencing profiles of forward strand using PCR product from the cDNA of: **b** non-carrier (control), **c-d** c.868-2A > G carriers, **e** non-carrier (family member)

To address if c.868-2A > G affects splicing, RT-PCR analysis of RNA extracted from two variant carriers and two non-carriers (one family member and one control) revealed the presence of one transcript corresponding to the reference full-length transcript (364 bp) in all samples (Fig. 2a). All transcripts were confirmed by Sanger sequencing (Fig. 2b-e). Thus, this variant may not affect the splicing of *RECQL.* It had a Sherloc score of 1P and 8B and was classified as benign (Table 4).

The remaining eleven variants (three missense variants and eight noncoding variants) have been previously reported as benign/likely benign in the ClinVar database (by April 2020) or in other populations.

## Discussion

This is the first study that investigates the prevalence of pathogenic *RECQL* germline variants in 302 *BRCA1* and *BRCA2* negative high-risk patients with ER positive and/or PR positive breast tumors from Pakistan. We identified a single novel pathogenic *RECQL* variant. Although several studies had been previously conducted in Europe and only two studies in East Asia, there is still conflicting evidence for a role of *RECQL* in breast cancer predisposition [6, 8, 10, 23]. Our study provides additional information on the contribution of the *RECQL* gene to hereditary breast cancer in a South Asian population from Pakistan.

The novel pathogenic *RECQL* variant, p.W75* was identified in 0.3% of early-onset and familial breast cancer patients with hormone receptor-positive tumors, but not in controls, suggesting that p.W75* may be disease-causative. In other studies performed in China [7, 11], higher pathogenic variant frequencies ranging from 0.54 to 1.6% were observed in *BRCA1* and *BRCA2* negative early-onset and/or familial breast cancer cases. In Caucasian studies conducted in the Australia [10], Canada [6], Poland [6], and USA [9], similar variant frequencies ranging from 0.1 to 0.4% have been reported in familial breast cancer patients, while no pathogenic variants were detected in studies performed in South-West Poland and West Ukraine [24]. In other Caucasian studies conducted in Belarus, Germany, and Australia, the frequency of pathogenic variants identified in controls were similar or higher than cases [8, 10]. Overall, these findings suggest a controversial role of *RECQL* as a breast cancer susceptibility gene.

Previously, a missense variant (p.R215Q) in the highly conserved RecA-like domain D1 of RECQL (amino acid residues 63 to 281) is reported to disrupt the *RECQL* helicase activity and classified as a pathogenic mutation [7]. In the current study, a novel missense variant, p.I141F, in the same domain was found in one familial breast cancer patient (0.3%), but not in controls. It may also affect the ATP-dependent translocation activity of RECQL leading to disruption of helicase activity [25]. However, functional assays are warranted to confirm this finding. Nevertheless, the population allele frequency of p.I141F was rare among South Asians in the gnomAD. Overall population data, variant type, clinical observation and findings from in silico predictions suggest that p.I141F may be a VUS based on the Sherloc guidelines.

The recurrent splice-site variant, c.868-2A > G, was identified in three breast cancer patients (1.0%) and two controls (0.8%). Its similar frequency in cases and controls indicates that this variant may be benign. This is supported by the fact that it has a very high frequency (0.5669%) among South Asians in the gnomAD. In addition, RT-PCR analysis revealed that it did not affect the *RECQL* splicing. Thus, based on the Sherloc variant classification guidelines, our data suggest that c.868-2A > G may be benign. However, we cannot exclude that the aberrantly spliced allele may have escaped from detection due to the nonsense-mediated decay or other splicing events may have occurred that were not investigated in the present study.

The ER and PR positive breast tumor of the Pakistani patient with the pathogenic *RECQL* variant showed high grade and IDC histology. These findings are in line with those from other studies conducted in China [7], Poland [6], Belarus, and Germany [8] further supporting the notion that high grade, hormone receptor-positive breast tumors of IDC histology may be predictors of the pathogenic *RECQL* variant status.

Our study has several limitations. First, despite its reasonable size, larger studies are warranted to confirm our findings. Second, mutation analysis was restricted to patients with ER and/or PR positive breast tumors, in whom a predominance of pathogenic *RECQL* mutations has been reported [6–8, 11]. However, since patients with both ER and PR negative or triple-negative breast tumors were not tested, this may have undermined the prevalence of pathogenic *RECQL* variants reported in this study. Further, the functional analyses of the splice-site variant should be extended in order to confirm its classification as benign.

## Conclusion

In summary, we identified a single pathogenic *RECQL* variant in 302 *BRCA1* and *BRCA2* negative high-risk patients with ER positive and/or PR positive breast tumors. The frequencies of the novel pathogenic variant were 0.3% (1/302) in early-onset and familial breast cancer patients and 0.8% (1/133) in familial patients. Our data suggest that pathogenic *RECQL* variants explain a negligible proportion of hereditary breast cancer in Pakistan.

## Data Availability

The data stated in this article including clinical and phenotypic characteristics of breast cancer families are confidential. Data are available at the Laboratory of Basic Sciences Research supervised by Dr. Muhammad Usman Rashid (usmanr@skm.org.pk), for researchers who meet the criteria to access confidential data. All information that support our findings are included within this article.

## Abbreviations

ACMG-AMP: American College of Medical Genetics and Genomics-Association for Molecular Pathology
DHPLC: Denaturing high-performance liquid chromatography
ER: Estrogen receptor
gnomAD: Genome Aggregation Database
IDC: Invasive ductal carcinoma
IRB: Institutional Review Board
MAF: Minor allele frequency
PR: Progesterone receptor
*RECQL*: RecQ Like Helicase
RT-PCR: Reverse transcriptase polymerase chain reaction
SKMCH&RC: Shaukat Khanum Memorial Cancer Hospital and Research Centre
VUS: Variants of uncertain significance
