# Correction to: Prevalence of *RECQL* germline variants in Pakistani early-onset and familial breast cancer patients

**DOI:** 10.1186/s13053-020-00163-w

**Published:** 2021-01-07

**Authors:** Muhammad Usman Rashid, Noor Muhammad, Faiz Ali Khan, Umara Shehzad, Humaira Naeemi, Naila Malkani, Ute Hamann

**Affiliations:** 1 Department of Basic Sciences Research, Shaukat Khanum Memorial Cancer Hospital and Research Centre (SKMCH&RC), 7A, Block R3, Johar Town, Lahore, Punjab 54000 Pakistan; 2 Molecular Genetics of Breast Cancer, German Cancer Research Center (DKFZ), Im Neuenheimer Feld 580, 69120 Heidelberg, Germany; 3 Department of Zoology, Government College University, Lahore, Pakistan

**Correction to: Hered Cancer Clin Pract 18, 25 (2020)**

**https://doi.org/10.1186/s13053-020-00159-6**

Following publication of the original article [1], a typesetting error was identified. Figure 1 was not published in full. The complete Fig. 1 is given in this correction article and the original article [1] has been corrected.

**Fig. 1:**
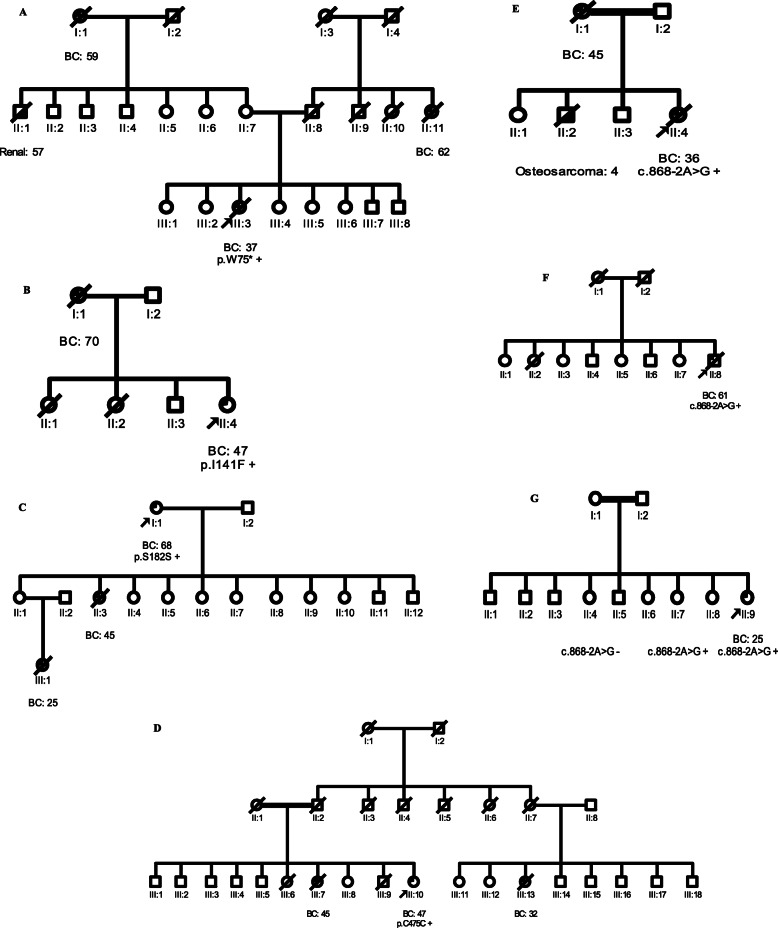
Pedigrees of breast cancer patients with *RECQL* variants. **a** Family 282 carrying the pathogenic variant p.W75*. **b-d** Families 565, 649, and 625 carrying the VUS p.I141F, p.S182S, and p.C475C, respectively. **e-g** Families 471, 577 and 595 carrying the benign variant c.868-2A > G. *Circles* are females, *squares* are males, and a *diagonal slash* indicates a deceased individual. *Symbols* with filled *left upper* quadrant: unilateral breast cancer. *Symbols* with *filled right lower* quadrant: cancer other than breast, the name of that cancer is indicated. *Double line* between spouses: consanguineous marriage. Identification numbers of individuals are below the *symbols*. The index patient is indicated by an *arrow*. *BC:* breast cancer. The numbers following these abbreviations indicate age at cancer diagnosis. +: carrier, −: non-carrier
